# Factor VII-Activating Protease (FSAP) and Its Importance in Hemostasis—Part II: A Link Between FSAP, Blood Coagulation, and Fibrinolysis: A Narrative Review

**DOI:** 10.3390/ijms262110709

**Published:** 2025-11-03

**Authors:** Iga Schachta, Ewa Żekanowska, Jan Styczyński, Joanna Murawska, Simona Lattanzi, Andrea M. Alexandre, Artur Słomka

**Affiliations:** 1 Department of Pathophysiology, Nicolaus Copernicus University in Toruń, Ludwik Rydygier Collegium Medicum, 85-094 Bydgoszcz, Poland; iga.schachta@cm.umk.pl (I.S.); zorba@cm.umk.pl (E.Ż.); joanna.murawska@doktorant.umk.pl (J.M.); artur.slomka@cm.umk.pl (A.S.); 2 Department of Paediatrics, Haematology and Oncology, Nicolaus Copernicus University in Toruń, Ludwik Rydygier Collegium Medicum, 85-094 Bydgoszcz, Poland; 3 Neurological Clinic, Department of Experimental and Clinical Medicine, Marche Polytechnic University, 60121 Ancona, Italy; alfierelattanzisimona@gmail.com; 4 UOSD Neuroradiologia Interventistica, Fondazione Policlinico Universitario A. Gemelli IRCCS Roma, 00168 Rome, Italy; andrea.alexandre@policlinicogemelli.it; 5 Department of Hematology and Oncology, National Medical Institute of the Ministry of Interior and Administration, 02-507 Warsaw, Poland

**Keywords:** factor VII-activating protease, coagulation, hemostasis

## Abstract

As a continuation of Part I on the structure and regulation of factor VII-activating protease (FSAP), this narrative review synthesizes mechanistic, translational, and limited clinical evidence to delineate FSAP’s roles at the interface of coagulation and fibrinolysis. Current evidence indicates that FSAP enhances thrombin generation primarily via proteolytic inactivation of tissue factor pathway inhibitor (TFPI), whereas direct activation of factor VII (FVII) by FSAP appears weak or context-restricted. Beyond plasma proteins, FSAP can upregulate tissue factor (TF) in human macrophages, while platelet-related effects remain insufficiently substantiated. On the fibrinolytic axis, FSAP indirectly accelerates clot lysis by converting single-chain urokinase (scuPA) to its active two-chain form (tcuPA) and, less efficiently, by processing tissue-type plasminogen activator (tPA); in addition, selective cleavage of fibrinogen Aα and Bβ chains remodels clot architecture, yielding thinner fibers with higher density and increased susceptibility to proteolysis. Collectively, the data position FSAP as a context-sensitive modulator of thrombin generation and fibrin turnover. Key gaps include isoform specificity, in vivo cellular targets, and the quantitative contribution of the FSAP-TFPI and FSAP–fibrinogen–urokinase/tPA axes in human pathophysiology, which warrant focused mechanistic and clinical studies.

## 1. Introduction

Factor VII-activating protease (FSAP) is a plasma protein first described by Choi-Miura et al. in 1996 [1]. In the literature, FSAP is also known as plasma hyaluronan-binding protein (PHBP) [1,2,3]. It is also called plasma hyaluronan-binding serine protease (PHBSP) [4] and hyaluronic acid binding protein 2 (HABP2) [5,6]. FSAP protein is encoded by the *HABP2* gene located on chromosome 10q25-q26 [2]. The main site of FSAP synthesis is the mouse and human liver [1,7].

In our first part of the manuscript, titled “Factor VII activating protease (FSAP) and its importance in hemostasis. Part I: FSAP structure, synthesis and activity regulation: a narrative review”, we focused in detail on the structure of FSAP and its synthesis sites [8]. We also discussed regulators of FSAP expression, FSAP activation process, and modulators of FSAP activity [8]. In this paper, we aim to demonstrate the numerous connections between FSAP and components of the coagulation and fibrinolysis.

This narrative review synthesized evidence on FSAP’s roles in coagulation and fibrinolysis through a comprehensive literature search. Relevant studies were identified using the PubMed database with the following search terms: “Factor VII activating protease,” “FSAP,” and “HABP2.” The search encompassed primarily original research articles, as well as review papers, all published in English. Studies were selected based on their relevance to FSAP’s influence on hemostasis.

## 2. The Role of FSAP in Hemostasis—A Multifunctionality

Many studies suggest the role of FSAP as a modulator of blood coagulation and fibrinolysis [3,4,6,9,10,11,12,13,14,15,16,17,18,19,20,21,22,23,24,25,26,27,28]. Initially, the prominent role of FSAP was associated only with the activation of coagulation factor VII (FVII) [9], which, in a complex with tissue factor (TF), initiates blood coagulation in vivo. The name of the protein, factor VII-activating protease (FSAP), is derived from these experiments [9]. Subsequent in vitro studies revealed that FSAP has other functions, including the degradation of tissue factor pathway inhibitor (TFPI) and its involvement in blood clot lysis. Therefore, FSAP likely plays diverse roles in hemostasis. This subsection highlights the multifunctionality of FSAP in blood coagulation and fibrinolysis.

### 2.1. Blood Coagulation

#### 2.1.1. TFPI: The Prominent Substrate of FSAP?

The procoagulant role of FSAP was originally attributed to its activation of FVII in vitro [4,9]. However, current data have shown that the propagation of coagulation by FSAP is associated with TFPI inhibition [10,11,12,13]. TFPI impedes coagulation by inactivating the complex of TF, activated factor X (FXa), and activated factor VII (FVIIa) [10,11,29]. Thus, FSAP, by inhibiting TFPI, may indirectly promote the generation of FVIIa and the formation of a blood clot.

##### FSAP-Mediated Regulation of TFPI Isoforms

TFPI consists of two main naturally occurring forms [10,11]. TFPI alpha (TFPIα) [10,11] is a full-length TFPI with all three Kunitz-type domains: Kunitz-type domain 1 (K1), Kunitz-type domain 2 (K2), and Kunitz-type domain 3 (K3) [10,11,29]. TFPIα is synthesized predominantly by human cells [10,11]. TFPI beta (TFPIβ) lacks K3 [10,11] and has an alternative C-terminal region [11]. TFPIβ is predominantly observed in adult mice [10,11]. FSAP inhibits TFPI by cleaving it [10,11] at multiple sites, such as between K1 and K2, as well as in the active sites of K2 and K3 [10]. FSAP binds to the C-terminal binding domain of TFPI [10,29], likely a critical step in TFPI inactivation [10]. According to Kanse et al., the reduction in TFPI activity by FSAP was ineffective without FSAP-TFPI binding [10]. For this reason, full-length TFPIα was initially expected to be a significantly better substrate of FSAP than the two-domain TFPIβ [10].

Although the data are fragmentary, there is a premise that FSAP may inhibit both TFPI isoforms [11]. This assumption is based on the FSAP-driven inhibition of TFPI [10,11] observed at sites that differ in the expression of TFPI isoforms. For instance, decreased TFPI expression in murine platelets by FSAP possibly indicates inhibition of TFPIα, the predominant form produced by these cells [11]. The following observations suggest that TFPIβ is inactivated by FSAP [11]. Firstly, FSAP inhibited TFPI derived from human umbilical vein endothelial cells (HUVECs) [10], probably the endothelial cell-associated form, i.e., TFPIβ [11]. Secondly, in an in vivo pulmonary embolism (PE) model, murine plasma TFPI levels and activity, most likely TFPIβ as it predominates in mice, depended on endogenous FSAP activity [11]. In summary, the molecular mechanism of TFPI inhibition by FSAP remains vague, as current data partly rely on assumptions about TFPI isoforms rather than their direct identification. The studies mentioned above provide a preliminary outline of the connections between FSAP, TFPIα, and TFPIβ; however, a more detailed experimental approach is required to draw definitive conclusions.

##### TFPI Inhibition by FSAP: Insights from In Vitro and In Vivo Data

Several studies have provided scientific evidence of TFPI inhibition by FSAP, beginning with in vitro experiments [10,12,13]. Reduced TFPI activity due to FSAP was observed in purified systems [10,12] and in HUVEC culture [10]. FSAP decreased TFPI levels and activity in HUVECs without affecting TFPI mRNA levels [10]. Exogenous FSAP reduced TFPI function in plasma by more than 50% [13] to 60% [10]. In TFPI-depleted plasma, FSAP did not alter diluted prothrombin time (dPT) despite normal FVII levels [13]; however, pretreatment of TFPI with FSAP accelerated dPT by 30–40 s [10]. FSAP increased plasma FVIIa levels significantly, but only in the presence of TFPI [13]. These findings demonstrate that FSAP can activate the extrinsic pathway of coagulation in vitro by inactivating TFPI, rather than directly activating FVII.

In vitro studies of the FSAP-TFPI-FVII relationship were corroborated by in vivo experiments in mice [11]. The association between FSAP, TFPI, and FVII in vivo was evident only under pathological conditions, i.e., after PE induction in wild-type (WT) and FSAP-deficient mice [11]. Compared to the FSAP-deficient strain with PE, the WT strain with PE exhibited lower total TFPI levels in plasma and lung tissue, reduced TFPI activity, and decreased FVII levels in plasma, but comparable FVIIa plasma levels [11]. The authors concluded that in the PE model, FSAP likely inhibited TFPI, leading to FVII consumption for the activation of coagulation [11]. Unexpectedly, FVII consumption did not correspond with increased FVIIa levels, which warrants further investigation. Furthermore, some in vivo models may generate negligible systemic effects, as in the case of carotid artery thrombosis in mice, which failed to present any relationship between endogenous FSAP, plasma TFPI, FVII, and FVIIa [11]. Only exogenous FSAP, administered before induced arterial thrombosis, affected TFPI plasma levels, reducing them in WT mice in comparison with FSAP-deficient mice [11]. Notwithstanding, Subramaniam et al. [11] provided a valuable contribution by demonstrating that the association between FSAP and TFPI exists in vivo and its potential impact on FVII levels.

**Figure 1:**
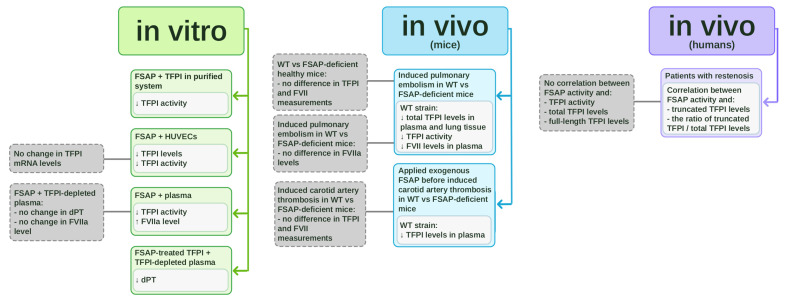
In vitro and in vivo studies on tissue factor pathway inhibitor (TFPI) inhibition by factor VII-activating protease (FSAP). The schematic summarizes experimental evidence on the interaction between FSAP and TFPI. In vitro studies (marked in green) include purified systems, HUVEC cultures, plasma assays, and experiments using FSAP-treated TFPI in TFPI-depleted plasma. In vivo studies in mice (marked in blue) involve comparisons between wild-type (WT) and FSAP-deficient animals in pulmonary embolism and carotid artery thrombosis models. In vivo clinical studies in patients with restenosis (marked in violet) demonstrate correlations between FSAP and TFPI levels.

Despite its potential importance, limited clinical data have assessed the relationship between FSAP and TFPI in humans. In patients with restenosis, FSAP activity correlated with truncated TFPI levels (r^2^ = 0.52, *p* = 0.03) [13]. The correlation was stronger when FSAP activity was compared with the ratio of truncated TFPI to total TFPI levels (r^2^ = 0.55, *p* = 0.02) [13]. No significant correlation was observed between FSAP activity and TFPI activity, total TFPI levels, or full-length TFPI levels [13]. Etscheid et al. suggested that FSAP activity might determine the extent of TFPI cleavage in vivo [13]. However, further research is needed to confirm this, given the pilot nature of the studies conducted so far. The in vitro and in vivo studies on TFPI inhibition by FSAP are collected in Figure 1.

One study published in 2012 [29] stands in opposition to the in vitro and in vivo reports on TFPI inhibition by FSAP. Stephan et al. focused on an inverse relationship, where TFPI inhibited FSAP activity in vitro [29]. What is essential is that the authors briefly concluded that FSAP did not degrade TFPI in their experiment, because TFPI-induced inhibition of FSAP activity was similar over time, suggesting unaltered TFPI activity [29]. On the one hand, this interpretation raises doubts about the nature of the relationship between FSAP and TFPI, which may need to be reexamined. On the other hand, the limited evidence excluding FSAP as the TFPI [29] contrasts with the growing body of literature supporting TFPI inhibition by FSAP [10,11,12,13]. Therefore, we remain inclined to regard TFPI as a substrate of FSAP.

In summary, the relationship with TFPI underscores FSAP’s strong role in the extrinsic pathway of coagulation. By inhibiting TFPI, FSAP may promote the activation of FVII, ultimately contributing to fibrin clot formation. The results of the cited in vitro and in vivo studies emphasize the urgent need for further investigation of these relationships in humans.

#### 2.1.2. The Controversy Surrounding FSAP and FVII

In light of current knowledge, TFPI inhibition is the core of the procoagulant mechanism of action of FSAP. However, FVII activation independent of TF [4,9] used to be assumed as the key function of FSAP, from which the protein was named. Early studies showed that FSAP activated FVII in the purified system [4,9]. However, with the emergence of new research, the view of the FVII and FSAP relationship has evolved, as described below.

Etscheid et al. reported that FSAP, even at levels close to physiological, activated FVII with high efficiency in human plasma; however, they attributed this observation to TFPI inhibition [13]. The authors conducted experiments [13], which, together with several independent studies [10,12,14], have shown that FVII is a weak substrate of FSAP in vitro. According to Etscheid et al., in a purified system, WT FSAP generated no more than 0.6% of FVIIa [13]. Kanse et al. observed that a maximum of 4% of purified FVII can be activated by FSAP, but only at supraphysiological levels of this protease [10]. In vitro, FVII activation required FSAP levels approximately 100 times higher than those required for the activation of another FSAP substrate, urokinase plasminogen activator (uPA) [12]. Compared to single-chain uPA (scuPA), FVII was cleaved by isolated FSAP with a sensitivity at least 50 times lower [13]. Stavenuiter et al. demonstrated that recombinant FSAP, even when added in a large excess over FVII, could not cleave FVII in vitro [14]. In a purified system, FXa formed FVIIa at least 1000 times faster than FSAP [14]. A comparable degree of FVII activation in vitro required 20 times less factor X (FX) than FSAP [13]. Thus, depending on the experiment, FVII activation required very high levels of purified FSAP or was not efficient at all. Studies in cell cultures confirmed the weak relationship between FSAP and FVII activation. According to the unshown data from Kanse et al., the addition of FSAP to HUVECs expressing TF caused an increase in FXa levels, but only when FVIIa and FX were added together with FSAP to the mixture [10]. This indicated that FSAP was unlikely to activate FVII or FX directly [10]. The above observations question the initial assumption that direct activation of FVII underlies the procoagulant property of FSAP.

Intriguingly, FSAP’s ability to activate FVII may depend on the surface on which this process occurs. FVII was not effectively activated by FSAP, even in the presence of recombinant TF or phospholipid vesicles containing phosphatidylserine (PS) and calcium ions [14]. However, the anionic phospholipid cardiolipin served as a surface that enabled FSAP to activate FVII in vitro [14]. This activation was followed by the rapid degradation of FVIIa [14]. Extrapolating this result to in vivo conditions is challenging, as the process requires membranes composed entirely of cardiolipin, which is not typically found in the blood [14]. Since cardiolipin is a component of the inner mitochondrial membrane, it could potentially be released during cell damage, providing a shared surface for FSAP and FVII activation [14]. Further research is needed to verify this hypothesis.

#### 2.1.3. FSAP and TF

According to the cellular model of coagulation [30], the complex of TF and FVII initiates blood clotting. FSAP does not have this capability [3]. While several papers have analyzed the association between FSAP and FVII, the connection with TF has been addressed only in a few in vitro studies, yielding inconclusive results [10,31]. FSAP was found to elevate TF mRNA and protein expression in human monocyte-derived macrophages [31], while having no effect in HUVECs [10]. These findings suggest that the influence of FSAP on TF may vary depending on the cell type studied in vitro. Additionally, Kanse et al. proposed that FSAP may play a role in TF signaling functions [10], but this hypothesis remains unexplored.

By stimulating TF expression, FSAP could exert a potential procoagulant effect. Targeted research into FSAP’s differential effects on TF across cell types could offer new perspectives on its functions in coagulation. It may be worth considering such tests on platelets in the face of debate on their ability or inability to synthesize TF [30] and platelet expression of FSAP [20].

#### 2.1.4. FSAP and Platelets: Is There a Link?

The relationship between FSAP and platelets has not been fully explored. Nonetheless, this association seems particularly interesting due to FSAP expression in platelets [20]. This expression was upregulated in vitro at the mRNA levels in response to platelet activators such as adenosine diphosphate (ADP) and thrombin receptor-activating peptide (TRAP) [20]. The same study showed that acetylsalicylic acid (ASA), an inhibitor of platelet activation and aggregation, reduced FSAP expression in platelets [20]. Furthermore, certain modulators of FSAP mRNA expression, protein levels [32], or activity [33] are stored in platelet granules [34,35]. These modulators include transforming growth factor-β (TGF-β) [32] and plasminogen activator inhibitor-1 (PAI-1) [33]. Further research is necessary to clarify the mechanisms linking FSAP expression and platelet function.

In screening and specialized platelet tests, no associations with FSAP have been identified [4,11,36]. Under normal conditions, endogenous FSAP activity in mice did not affect basic assays such as platelet count or mean platelet volume (MPV) [11]. FSAP also showed no effect on specialized in vitro platelet tests like platelet aggregation [4]. Similarly, platelet adhesion, activation, aggregation, and glycoprotein levels were comparable in WT and FSAP-deficient mice [11]. Studies of vascular injury in mice revealed no connection between endogenous FSAP activity and either platelet count [36] or platelet adhesion at the site of injury [11]. Therefore, based on current evidence, the only established link with FSAP is its expression in platelets.

#### 2.1.5. Other Coagulation-Related Factors

Studies on other coagulation-related parameters have mostly yielded contradictory or unclear results or have shown no association with FSAP. One study from 1999 reported that FSAP inactivated factor V (FV) and factor VIII (FVIII) in vitro [9]. However, this finding was not corroborated in vivo when clotting activities of these factors were assessed in mice [11]. Although no clear in vitro relationship was established, factor II (FII), FV, and kallistatin (a kallikrein inhibitor) were co-immunoprecipitated with FSAP antibody from human plasma [37]. Notwithstanding, these results may be biased by the secondary binding of various plasma proteins forming a macromolecular complex [37].

For the following parameters, no relationship to FSAP has been proven. FSAP did not alter anionic phospholipid content on the endothelial cell surface and did not affect thrombomodulin (TM) mRNA levels [10]. Moreover, FII [3,21], factor IX (FIX) [21], FX [10,21], factor XI (FXI), factor XII (FXII) [38], factor XIII (FXIII) [17], and plasma prekallikrein (PPK) [38] were shown not to be substrates of FSAP in vitro. Consistent with in vitro observations, the plasma activities of FII, FIX, FX, FXI, FXII, and PPK were independent of endogenous FSAP in mice [11].

Although TFPI seems to be a major FSAP substrate in blood coagulation, alternative mechanisms may also exist. Findings of Sperling et al. suggest a procoagulant function of FSAP through a TFPI-independent mechanism [22]. The incubation of human whole blood on a positively charged polyethylenimine (PEI) surface induced blood coagulation, possibly involving FSAP [22]. PEI promoted the activation of FSAP in plasma and whole blood [22]. FSAP neutralization in vitro reduced clot formation on the PEI surface [22]. These findings may provide valuable insights into the mechanisms of cationic surface-mediated coagulation, a process less studied than coagulation on anionic surfaces and its relation to contact phase factors. Further exploration of the link between PEI, FSAP, and blood clotting may lead to novel strategies for enhancing the hemocompatibility of PEI-based medical products [22]. Emerging evidence for a TFPI-independent mechanism of blood coagulation opens new avenues for research into FSAP’s biological significance.

### 2.2. Fibrinolysis

#### 2.2.1. The Association of FSAP with Fibrinolysis Activators

In contrast to its procoagulant properties, it is widely recognized that FSAP influences clot lysis in vitro. Although FSAP cannot initiate fibrinolysis in vitro [3,17], it influences plasminogen activators: uPA and tissue-type plasminogen activator (tPA), which cleave plasminogen to plasmin, allowing the breakdown of the fibrin clot [39]. FSAP converts the inactive scuPA into the active two-chain uPA (tcuPA) [3,6,11,12,13,14,15,19,20,21,22,23,24,25,26,27,28]. FSAP is a more effective activator of scuPA than kallikrein or FXIIa in vitro [15]. A connection between FSAP overexpression and elevated uPA activation was demonstrated in human lung adenocarcinoma cell cultures [5]. In the study by Kannemeier et al., uPA enhanced the autoactivation of FSAP in vitro [23]. Thus, FSAP and uPA may likely interact in a mutual up-regulatory mechanism, amplifying each other’s activation.

The ability of FSAP to activate scuPA is commonly used to determine FSAP activity in numerous studies [6,11,12,13,19,20,21,22,23,24,25,26,27,28,37]. Notwithstanding, it remains unclear whether relying solely on the scuPA-activating capacity is sufficient to evaluate overall FSAP activity [24,25,26]. It has been argued that measuring FSAP-driven uPA activation may not account for other FSAP functions, such as its procoagulant property [24,26]. There is also a risk of obtaining inconsistent FSAP activity results depending on the substrate used in the test [25,26].

The coincubation of FSAP with scuPA resulted in rapid fibrin lysis in the whole blood thromboelastography (TEG) assay, whereas scuPA alone presented no significant effect [15]. Römisch et al. implied that FSAP was required to activate scuPA, which subsequently initiated fibrinolysis [15]. A similar conclusion was drawn by Semeraro et al. [16]. Namely, free histones enhanced FSAP autoactivation, leading to the activation of scuPA in plasma, thereby shortening scuPA-driven clot lysis time in vitro [16]. Nevertheless, the involvement of FSAP in accelerating clot lysis may follow a different mechanism unrelated to scuPA activation. Etscheid et al. showed that FSAP did not increase scuPA-driven clot lysis in a fibrin plate assay [17]. According to their study, the faster clot lysis induced by FSAP was more likely linked to fibrinogen alterations rather than to scuPA activation [17].

Compared to uPA, the role of FSAP concerning another plasminogen activator, tPA, is less well understood. Isolated FSAP enhanced the activation of single-chain tPA (sctPA) to two-chain tPA (tctPA) in vitro [3,15]. Notably, sctPA was a less efficient substrate for FSAP compared to scuPA [15]. Subsequent observations [17,27] raise doubts about the significance of the interaction between tPA and FSAP. Etscheid et al. reported that exogenous FSAP, unlike exogenous tctPA, failed to initiate clot lysis in plasma [17]. This observation implied that FSAP probably did not activate enough sctPA in plasma [17]. As previously mentioned, FSAP shortened clot lysis in vitro, but this effect was independent of plasminogen activators and instead relied on fibrinogen modification [17]. Furthermore, no correlation was found between tPA levels and FSAP plasma levels or activity in healthy human subjects [27]. This study [27] did not specify whether sctPA, tctPA, or both forms were measured. Based on the current data, it cannot be clarified whether FSAP significantly regulates tPA levels in vivo.

#### 2.2.2. The FSAP-Driven Changes in Fibrinogen

Fibrinogen, known as coagulation factor I (FI), is cleaved by thrombin to produce fibrin [40]. Fibrin then polymerizes to form the structural scaffold of a blood clot, which is ultimately degraded by plasmin [40]. Although fibrinogen is involved in blood coagulation, its FSAP-driven modifications are associated with increased clot susceptibility to lysis in vitro [17].

FSAP binds strongly to immobilized fibrinogen [37]. Fibrinogen consists of two subunits, each containing three polypeptide chains: Aα, Bβ, and γ [17]. The structure of fibrinogen includes several globular regions: a central E region, two lateral D regions, and two αC regions [17]. FSAP specifically targets the Aα and Bβ chains of fibrinogen [3,17]. In vitro, FSAP cleaves the Aα-chain [3,17] at multiple sites, partially truncating the αC region [17]. FSAP also cleaves the Bβ-chain [3,17], releasing the N-terminal peptide Bβ(1–53) [17]. This is followed by the release of fibrinopeptide B (FpB) [17]. Therefore, the effect of FSAP on fibrinogen differs markedly from the cleavage patterns of thrombin and plasmin.

FSAP-induced alterations in the fibrinogen molecule are significant at the stage of clot formation and lysis. Fibrin, derived from FSAP-treated fibrinogen, exhibited delayed polymerization in vitro [17]. This polymerization resulted in a less coarse network of thinner fibrin fibers [17]. The formed clot had a denser structure, smaller pore size, and decreased turbidity, but it was susceptible to faster lysis in vitro [17]. The exact cause of the clot’s susceptibility to lysis is unknown. FSAP may modify fibrinogen structure and alter clot characteristics in vivo.

In healthy individuals, fibrinogen levels positively correlate with plasma FSAP levels (r = 0.15, *p* < 0.001) [27], (r = 0.26, *p* < 0.001) [28]. A similar correlation is observed with fibrinogen levels and FSAP activity (r = 0.17, *p* < 0.001) [27], (r = 0.28, *p* < 0.001) [28]. These data may result from potential in vivo interactions. Considering FSAP’s impact on clot structure and susceptibility to lysis in vitro, analyses in animal models and patients are warranted to verify potential therapeutic applications of FSAP.

#### 2.2.3. FSAP and Other Fibrinolysis-Related Factors

Some studies mention the links between FSAP and other fibrinolysis-associated proteins. Plasminogen is a zymogen of plasmin, which degrades fibrin and fibrinogen [39]. Two papers mentioned that FSAP did not directly influence plasminogen activation [3,15]. However, FSAP was co-immunoprecipitated with plasminogen from normal human plasma and bound strongly to immobilized plasminogen [37]. FSAP can also indirectly generate plasmin through uPA and tPA activation [15]. Once activated by FSAP, uPA and tPA induce plasmin formation from plasminogen, giving FSAP potential indirect pro-fibrinolytic capacity.

The inhibitors of the fibrinolytic system are widely known, including PAI-1, which neutralizes uPA and tPA [39]. PAI-1 has been shown to inhibit FSAP activity in vitro [33]. Moreover, in healthy humans, plasma PAI-1 levels positively correlated with FSAP levels (r = 0.16, *p* < 0.001) and FSAP activity (r = 0.11, *p* = 0.006) [27]. These correlations may reflect PAI-1’s inhibition of FSAP. However, another causation cannot be excluded at this stage, as the connection between FSAP and PAI-1 is poorly understood.

Another fibrinolysis inhibitor is the thrombin-activatable fibrinolysis inhibitor (TAFI). TAFI converts to its active form, TAFIa, due to the activation by thrombin, plasmin, or the thrombin/thrombomodulin complex [41]. This activation releases the N-terminal activation peptide, a component of TAFI structure [41]. In healthy humans, FSAP levels were positively correlated with TAFI (r = 0.17, *p* < 0.001) and TAFI activation peptide (r = 0.16, *p* < 0.001) [27]. Similarly, FSAP activity positively correlated with TAFI (r = 0.13, *p* < 0.001) and TAFI activation peptide (r = 0.12, *p* = 0.003) [27]. Therefore, FSAP correlates not only with TAFI but also with the marker of TAFI activation. While these correlations do not prove a direct relationship between FSAP and fibrinolysis inhibitors, they provide potential directions for future research.

### 2.3. Laboratory Tests of the Hemostatic System and Coagulation In Vivo in Mice

In this section, we present the functions of FSAP, focusing on its ability to modulate basic and often used laboratory tests that assess blood coagulation. These tests include activated partial thromboplastin time (aPTT) and prothrombin time (PT). PT reflects the status of the extrinsic pathway of coagulation and is partially dependent on FVII activity [18,42,43]. aPTT is used to assess the intrinsic pathway of the clotting cascade [42]. These tests are routinely performed in everyday clinical practice to evaluate blood coagulation [43].

The impact of FSAP on PT remains controversial [4,13,18,19]. Some authors reported that FSAP shortens PT [18] and diluted PT (dPT) in vitro [13,19], whereas one study showed no effect [4]. Etscheid et al. noted that only high FSAP levels over 200 nM shortened dPT [13]. At low FSAP levels, no effect was observed in vitro, likely due to neutralization of FSAP by plasma inhibitors [13]. Additionally, conflicting PT findings may be partially explained by the relationship between FSAP, FVII, and TFPI. Initially, FSAP was considered an activator of FVII, but it was later identified as an inhibitor of TFPI, which degrades the TF-FXa-FVIIa complex. This inhibition of TFPI could potentially result in higher FVIIa activity and thus shorter PT values, but the alterations in TFPI activity may not always be sufficient to affect PT [44]. Thus, discrepancies in the effect of FSAP on PT may arise from its TFPI-dependent regulation of FVII activity.

It has been demonstrated that FSAP did not affect aPTT [4,18]. This lack of effect on aPTT is expected, as FSAP does not interact with coagulation factors that influence aPTT.

Equivocal results have been obtained regarding the effect of FSAP on the results of viscoelastometric clot measurement in TEG [11,15]. Römisch et al. demonstrated that isolated human FSAP slightly shortened the reaction time, suggesting accelerated coagulation [15]. In contrast, Subramaniam et al. found no connection between TEG results and endogenous FSAP activity in mice [11]. Thus, the impact of FSAP on TEG results remains inconclusive.

The contribution of FSAP to coagulation has also been investigated in murine models. While the endogenous activity of FSAP in mice was not relevant for the results of bleeding time (BT) in the tail bleeding test, FSAP-deficient animals showed a tendency for rebleeding and increased total blood loss [11]. Following induced arterial damage, FSAP-deficient and wild-type (WT) mice exhibited similar platelet responses and times of initial thrombus formation [11]. However, FSAP deficiency led to delayed and reduced formation of stable thrombi [11]. These in vivo findings confirm that FSAP is involved in secondary hemostasis, which is controlled by coagulation factors, rather than the primary hemostasis reliant on endothelial and platelet function. The above-mentioned observations in mice highlight the importance of FSAP in promoting blood coagulation rather than fibrinolysis.

Therefore, in vitro experiments and in vivo models indicate that the procoagulant functions of FSAP, such as TFPI inhibition, could theoretically outweigh its potential effects on fibrinogen modifications or the activation of plasminogen activators. This assumption is supported by the observation that uPA, a recognized substrate of FSAP, exerts only a limited influence on fibrinolysis activation, as it lacks a fibrin-binding domain and acts largely in a fibrin-independent manner [45]. In contrast, tPA displays high fibrin affinity [45]; however, its interaction with FSAP remains poorly understood and appears to depend on experimental conditions.

Moreover, in theory, the procoagulant actions of FSAP may suppress the functionality of plasminogen activators through downstream effects. FSAP promotes blood coagulation, i.e., thrombin generation, which may possibly lead to greater FXIII activation [45]. This could result in stronger fibrin cross-linking and enhanced incorporation of α-2-antiplasmin into the developing secondary plug [40,45], thereby increasing clot resistance to fibrinolysis and limiting the activity of tPA and uPA [45].

Furthermore, FSAP-induced thrombin generation possibly promotes TAFI activation, which reduces plasminogen accessibility for plasminogen activators [45]. This hypothesis is supported by observed correlations between FSAP levels and TAFI, as well as with TAFI activation markers, in healthy individuals. In summary, by promoting thrombin formation, FSAP may indirectly enhance clot stability and confer protection against fibrinolysis.

Nevertheless, according to the current state of knowledge, FSAP’s impact on basic and advanced laboratory parameters of blood coagulation remains ambiguous and requires further investigation with well-designed research models.

## 3. Conclusions

**Figure 2:**
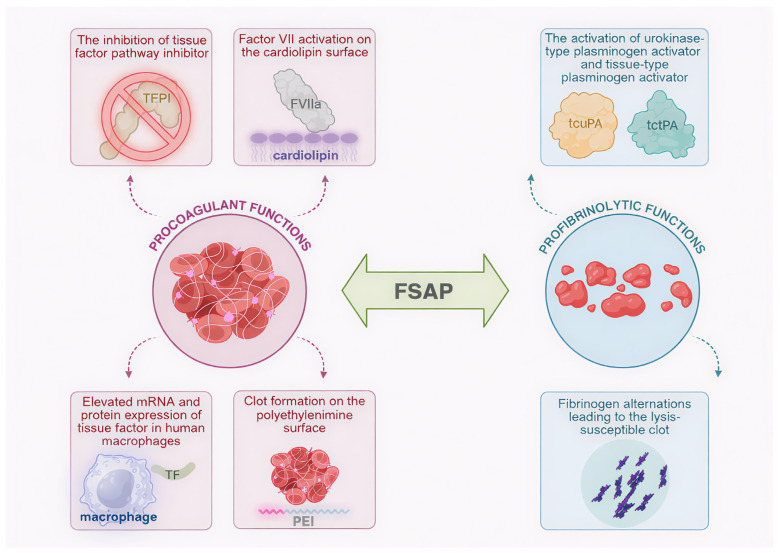
Multifunctionality of factor VII-activating protease (FSAP): schematic representation of FSAP involvement in clot formation and lysis. Procoagulant functions include the activation of factor VII (FVII) to active factor VII (FVIIa) in the cardiolipin surface, elevated messenger RNA (mRNA) and protein expression of tissue factor (TF) in human macrophages, the inhibition of tissue factor pathway inhibitor (TFPI), and clot formation on polyethylenimine (PEI) surface. Profibrinolytic activities are represented by fibrinogen alterations leading to the lysis-susceptible blood clot as well as the activation of urokinase plasminogen activator (uPA) and tissue-type plasminogen activator (tPA) to two-chain uPA (tcuPA) and two-chain tPA (tctPA), respectively. Created in Biorender. Iga Schachta and Artur Słomka. (2025), https://BioRender.com.

This paper shows FSAP as a promising factor in both coagulation and fibrinolysis. Its many hemostasis-associated roles are illustrated in Figure 2.

Part II describes the multifunctionality of FSAP in terms of the formation and lysis of blood clots. Most of the mentioned studies were conducted in vitro. At present, it is not possible to definitively determine which of the many different capabilities, and under which circumstances, may predominate in hemostasis in humans. Part III covers the inflammation-associated functions of FSAP and FSAP polymorphisms and collects data on the clinical significance of FSAP in human diseases.

## Data Availability

Not applicable.
